# Constructing Artificial Features with Grammatical Evolution for the Motor Symptoms of Parkinson’s Disease

**DOI:** 10.3390/bioengineering12121318

**Published:** 2025-12-02

**Authors:** Aimilios Psathas, Ioannis G. Tsoulos, Nikolaos Giannakeas, Alexandros Tzallas, Vasileios Charilogis

**Affiliations:** Department of Informatics and Telecommunications, University of Ioannina, 451 10 Ioannina, Greece; pint00141@uoi.gr (A.P.); giannakeas@uoi.gr (N.G.); tzallas@uoi.gr (A.T.); v.charilog@uoi.gr (V.C.)

**Keywords:** machine learning, evolutionary algorithms, genetic programming, grammatical evolution

## Abstract

People with Parkinson’s disease often show changes in their movement abilities during the day, especially around the time they take medication. Being able to record these variations in an objective way can help doctors adapt treatment and follow disease changes more closely. A methodology for quantitative motor assessment is proposed in this work. It employs data from a custom SmartGlove equipped with inertial sensors. A multi-method feature selection scheme is developed, integrating statistical significance, model-based importance, and variance contribution. The most significant features were retained, and higher-level artificial features were generated using Grammatical Evolution (GE). The framework combines multi-criteria feature selection with evolutionary feature construction, providing a compact and interpretable representation of motor behavior. Additionally, the framework highlights nonlinear and composite features as potential digital biomarkers for Parkinson’s monitoring. The method was validated on recordings collected from Parkinson’s patients before and after medication intake. The recordings have been retrieved during four standardized hand motor tasks targeting tremor, bradykinesia, rigidity, and general movement anomalies. The proposed method was compared with five existing machine learning models based on artificial neural networks. GE-based features reduced classification errors to 10–19%, outperforming baseline models. Furthermore, the proposed methodology performs prediction and recall 80–88%.

## 1. Introduction

Global health systems face a significant and growing challenge in the form of neurodegenerative diseases. These conditions cause severe motor and cognitive impairment and are characterized by a progressive disruption of the structure and function of neurons. As the world’s population ages, neurodegenerative diseases like Parkinson’s and Alzheimer’s disease are becoming more common. These conditions have a significant negative impact on patients’ quality of life and are becoming more and more expensive. Innovative methods of early detection, assessment, and treatment are required for these difficult disorders.

A common neurodegenerative disease that primarily affects the motor system is Parkinson’s disease (PD). Tremor, bradykinesia (slow movement), rigidity, and postural instability are classic clinical presentations. Non-motor symptoms like mood disorders, sleep disorders, and cognitive impairment are frequently present as well. Since there are no particular biological tests that can result in an early diagnosis, PD diagnosis primarily depends on clinical evaluation, despite the fact that the condition’s symptom profile is complex. As a result, slow symptom progression leads to misdiagnosis or delay, which leads to ineffective treatment, poor patient outcomes, and a lower quality of life [1].

The early detection and monitoring of PD could be greatly aided by recent technological advancements, particularly in the fields of machine learning (ML) and the internet of things (IoT). IoT sensors such as smartwatches, wearable health monitors, and others allow continuous and non-intrusive monitoring of motor activities, tremor frequencies, and gait abnormalities in real time. These tools enable an evaluation of a patient’s health while they are engaging in their regular activities and continue to gather large amounts of data outside of the typical clinical settings. One can more accurately identify early indicators of PD and enable early interventions by using machine learning techniques on this data [2,3]. For example, ML algorithms can analyze speech patterns, motor skills, and other non-motor symptoms and differentiate subtle patterns that could foretell the full-blown expression of the disease.

The pursuit of accurate, quantitative, and real-time measurement of PD motor symptoms has been the prime interest of computational neurology. It is rich in the current state-of-the-art research that utilizes diverse sensor modalities and machine learning (ML) methodologies. Pioneering work has validated the potential of smartphone sensor-derived composite scores, like the mobile PD Score (mPDS), that highly correlate to the conventional standards, such as the MDS-UPDRS, and that are capable of tracking intraday symptom variation as well as response to therapy [4]. In the same line, smartphone-enabled active tests as well as passive monitoring during clinical trial protocols have been shown to be realistic, reliable, and highly sensible, usually uncovering abnormalities even among those patients rated as normal on specific items of the UPDRS during the clinic evaluation [5].

In addition to smartphones, specific PD symptoms have been targeted with special-purpose sensors. Quantification of bradykinesia, the PD hallmark, has been achieved successfully with gyrosensors during tapping tests, demonstrating strong correlations with clinical ratings [6]. Tremor measurement has been automated with body-worn accelerometers combined with Hidden Markov Models, classifying tremor type and severity reliably [7]. Moreover, inertial sensors have been used to detect and classify complex gait disorders such as Freezing of Gait (FOG) reliably [8]. Even speech has been targeted in the remote monitoring paradigm, where off-the-shelf speech tests analyzed with signal analysis combined with regression models are capable of tracking PD progression with clinically helpful accuracy [9]. Notably, incorporation of the resulting objective measures into standard care has been shown to improve significantly the resulting clinical outcomes, including the UPDRS as well as quality-of-life scores [10]. Latest advances still investigate the integration of sensor data and advanced ML. Research has looked into the integration of speech signals along with ensemble classifiers such as AdaBoost [11] as well as the processing of gait as well as tremor data obtained from off-the-shelf wearable devices. Still missing, though, is an integrative, high-fidelity method for tracking the subtle kinematics of hand movement, a key domain for the measurement of bradykinesia as well as tremor beyond controlled smartphone tasks.

This work contributes to this vibrant body of research through the proposal of a unifying methodology grounded in a bespoke SmartGlove system that would allow in-depth analysis of hand movement. Our contribution is tripartite:

- Innovation and pilot use of the SmartGlove, a sensor-rich tool to capture high-accuracy, multi-parameter kinematic output of the hand.
- Construction of a holistic, multiple-component feature selection framework that goes beyond single technologies by integrating statistical significance (*t*-tests), ensemble learning (Random Forest), and analysis of variance (PCA) to pick the most discriminating, non-redundant biomarkers.
- The recognition of essential attributes especially nonlinear dynamics and spectral traits that are notably proficient in differentiating motor states in PD patients both prior to and following medication, offers an enhanced array of digital biomarkers for prospective diagnostic and monitoring applications.

By emphasizing the rich data space of hand kinematics and adopting a rigorous, multi-faceted analytical framework, this research hopes to improve the accuracy and completeness of objective PD evaluation. Briefly stated, the key innovation of this work lies in the combination of a specially crafted data-capture device with an upper-level analytical framework. Contrast this with previous work, which has employed repurposed consumer devices or targeted a very limited set of symptoms, and our specially developed SmartGlove presents a previously unmatched, high-fidelity view of hand motion’s rich kinematics. Coupled with our multi-modal feature choice methodology, this system does not merely replicate clinical scores but uncovers a novel class of physiologically enlightening biomarkers specifically in the nonlinear dynamic range that are hidden under conventional analysis. This end-to-end machine learning model is a significant advancement toward precise, data-based neurology from correlation against clinical scales to the detection of underlying digital biomarkers for PD.

In this work, extensive use was made of the Grammatical Evolution [12] technique to create artificial features from existing ones in order to significantly reduce the number of required inputs and to detect hidden correlations in the initial features of the patterns. The applications of the Grammatical Evolution includes cases such as function approximation [13,14], economic problems [15], network security issues [16], water quality problems [17], medical problems [18], evolutionary computing [19], temperature prediction in data centers [20], solving trigonometric problems [21], composing music [22], construction of neural networks [23,24], numerical problems [25], video games [26,27], energy issues [28], combinatorial optimization [29], cryptography [30], production of decision trees [31], problems that appear in electronic circuits [32], etc.

The remainder of this paper follows this organization: Section 2 outlines the materials and methods utilized with a detailed description of the system architecture of the SmartGlove system, the process of data acquisition related to individual exercises of the hands, and the overall feature extraction process. Section 3 details the multi-faceted feature choice strategy and the composite scoring system used to determine the best biomarkers. Also, a detailed discussion on the experimental results is provided in Section 4 and finally, Section 5 discusses implications of the research results, points out limitations of the study, and suggests directions of future research.

## 2. Materials and Methods

This section describes the complete methodology developed for the objective measurement of motor symptoms of PD. The approach integrates a custom-developed data acquisition system with an advanced computational protocol for converting raw sensor data into highly discriminative biomarkers. The analysis follows this outline: first, the SmartGlove system and data collection protocol used during special motor experiments are described; second, the multi-domain feature extraction approach is outlined; third, the multi-method scoring regimen developed for feature selection is presented; and, last, the keynote technique of Grammatical Evolution for synthesizing virtual features for improved classification performance, dimension reduction, and generalizability is presented.

### 2.1. SmartGlove System and Sources of Data

The system of interest here as a primary data source is the SmartGlove system, a specially developed wearable system that has been created with the intent of telemonitoring motor symptoms of PD patients. It has been created with the support of the Operational Program of the Epirus Region 2014–2020 and ESPA 2014–2020 with a view of increasing innovative health and biotechnology solutions. The system of SmartGlove has three primary subsystems:

1. Textile Glove: Made of durable, biocompatible materials, the glove has conductive areas on the palm and fingers that aid movement recognition. It is sweat-resistant and can be washed. It is also expected to last longer than 10 years.
2. System on Chip (SoC): The central processing unit encompasses a low-energy microcontroller that incorporates a Bluetooth 5.0 module, facilitating wireless data communication while utilizing minimal energy resources (with a typical operational range of 10–100 m, contingent upon the surrounding environment).
3. Sensors: The glove contains a multi-modal sensor suite:
4. Flex sensors to measure finger bending.
5. Contact sensors to detect finger-to-palm interaction.
6. A 9-axis Inertial Measurement Unit (IMU), composed of a 3-axis accelerometer, 3-axis gyroscope, and 3-axis magnetometer, to identify hands’ orientation and movement. IMU has outputs of up to a sampling rate of 100 Hz and a resolution of 16 bits.

The custom 9-axis IMU provides data with a resolution of 16 bits and a sampling rate of up to 100 Hz. All sensor modalities—IMU, flex, and contact—were sampled synchronously at 100 Hz by the onboard System-on-Chip (SoC).

The system is supplemented with a power management circuitry developed for lithium-polymer batteries, as well as with flash memory used for transient storage of data. The SmartGlove complies with the IEC 60601-1 medical electrical apparatus safety standard, thus ensuring reliability for medical applications. The SmartGlove sends data via Bluetooth to the mobPark mobile app supporting Android 6.0+ as well as iOS 11+. The app acts as an intermediary providing the possibility of managing the profile of the users, providing screen-level cues of exercises, as well as capturing sensor data at a sampling rate of 100 Hz. Then the captured data gets securely sent via an encrypted HTTPS connection to a centralized cloud platform like AWS or Azure for storage, processing, and analyzing purposes with a consideration of the General Data Protection Regulation (GDPR).

### 2.2. Data Collection Exercises

For the scope of this study, the analysis was conducted exclusively on data from the 9-axis IMU (accelerometer, gyroscope, and magnetometer). While the SmartGlove also incorporates flex and contact sensors, the IMU data provides a comprehensive foundation for capturing the core kinematic characteristics of tremor, bradykinesia, and overall movement dynamics targeted in our exercises. The integration of flex and contact sensor data is a key direction for future work to capture a more complete digital model of hand function.

**Figure 1:**
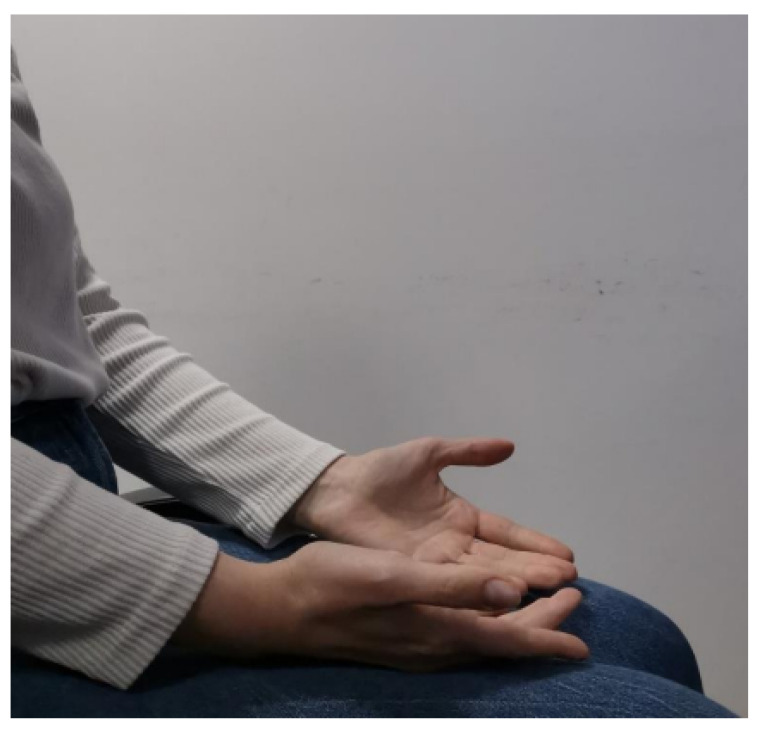
Resting Tremor Observation task. Illustration of the first exercise in which participants remained seated with their hands resting on their thighs, palms facing upward, allowing clear observation of resting tremor amplitude and rhythm under baseline conditions before or after medication.

**Figure 2:**
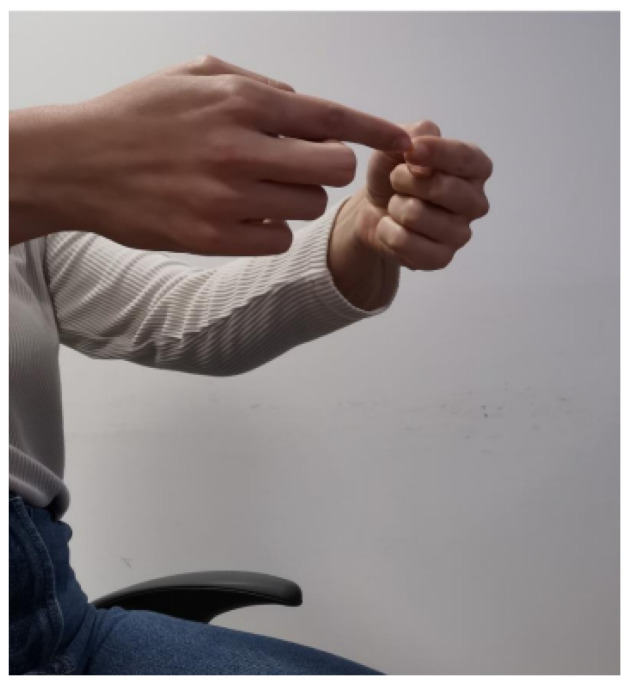
Postural Tremor and Coordination test. Depiction of the exercise used to evaluate postural tremor and hand–eye coordination, where subjects held their hands at shoulder height and attempted to touch their index fingers together while counting, revealing upper-limb stability and precision of movement.

**Figure 3:**
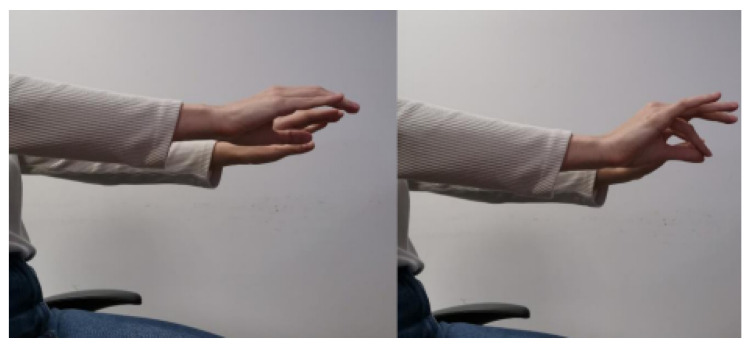
Finger Tapping Speed test. The participant performed ten rapid consecutive taps between the thumb and index finger, assessing bradykinesia and the speed of repetitive fine-motor actions characteristic of Parkinson’s disease.

**Figure 4:**
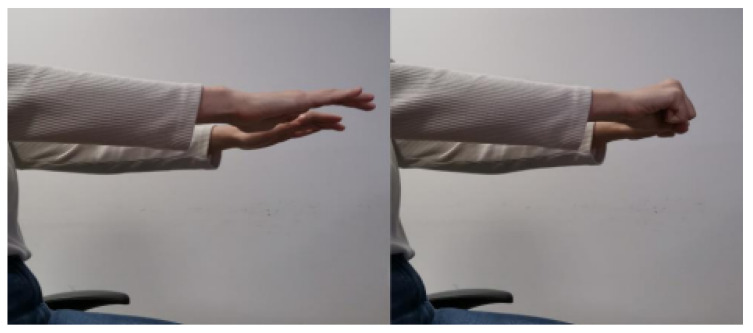
Hand Opening–Closing test. Illustration of the exercise in which the subject repeatedly performed full fist closures followed by full hand extensions, quantifying rigidity and bradykinetic movement through the amplitude and regularity of the motion.

The data recording at 100 Hz sampling frequency was selected for efficient tremor frequency (4–6 Hz) and bradykinesia capture. Prior to and after the administration of medicines, measurements were recorded in order for this therapeutic effect of medicines to be investigated. Four motor activities, according to standardized clinical examination, were performed:

- Exercise 0 (**Resting Tremor Observation**): For the evaluation of resting tremor (item 3.17 of the MDS-UPDRS), participants were seated in a chair, with hands resting in a supported position (on the arm-rests) and feet flat on the floor for 10 s according to the standard instructions. In our study, we adopted a slightly modified posture (hands resting on the thighs, palms facing upward) in order to align with the SmartGlove positioning protocol used for all participants. While this deviation from the canonical arm-rest position may impact comparability with other studies, it ensures consistent sensor placement and data acquisition across our cohort. An example of this exercise is shown in Figure 1.
- Exercise 1 (**Test for Postural Tremor and Coordination**): The patient as shown in Figure 2 held hands at shoulder height with fingers interlocked and attempted to touch their index fingers together after counting to ten. This evaluates postural tremor and coordination.
- Exercise 2 (**Finger Tapping Speed Test**): Lifting the palm, the subject made ten fast, repeated taps using the index finger on the thumb as shown in Figure 3. This is a routine measure for assessing motor speed and bradykinesia.
- Exercise 3 (**Hand Opening–Closing Test**): The patient did ten fast, sustained cycles of full fist then reopening the hand from a fully stretched palm position. This exercise evaluates hand rigidity and bradykinesia and an example of this exercise is shown in Figure 4.

### 2.3. Feature Extraction

Numerous features were taken from the raw sensor data (accelerometer, gyroscope, magnetometer) for each exercise in order to quantitatively describe the motor symptoms of Parkinson’s disease. These features were computed using 50% overlap (50 samples, 0.5 s) and sliding windows of 100 samples (1 s). The features extracted are as follows:

- Statistical Properties: These characteristics provide a succinct explanation of the signal’s variation and distribution. The following properties were calculated: Skewness, Kurtosis, Quartile Deviation, Mean, Standard Deviation, Variance, Minimum, Maximum, Range, Median, and Interquartile Range (IQR).
- Energy Characteristics: These metrics assess the signal’s intensity and degree of activity. Signal Magnitude Area (SMA), Root Mean Square (RMS), Total Energy, and Logarithmic Energy are among the features that were extracted.
- Frequency-Domain Features: These are essential for detecting tremors and take into account the signal’s spectral characteristics. Included are the following features: Dominant Frequency, Spectral Flatness, Spectral Flux, Spectral Variability, Spectral Entropy, Spectral Centroid, Spectral Spread, and Spectral Roll-on (85%).
- Dynamic and Nonlinear Features: These characteristics specify the signal’s temporal fluctuations, complexity, and predictability. The following characteristics are taken into consideration: Mean Absolute Deviation (MAD), Root Mean Square of Successive Differences (RMSSD), Higuchi Fractal Dimension, Lyapunov Exponent, and Sample Entropy.

Since each feature includes specific aspects of motor impairment typical of the disease, such as tremor regularity, movement amplitude, and signal complexity, the selection of features was driven by a large body of literature on PD analysis. The characteristics for movement analysis in Parkinson’s disease are outlined in Table 1.

**Figure 5:**
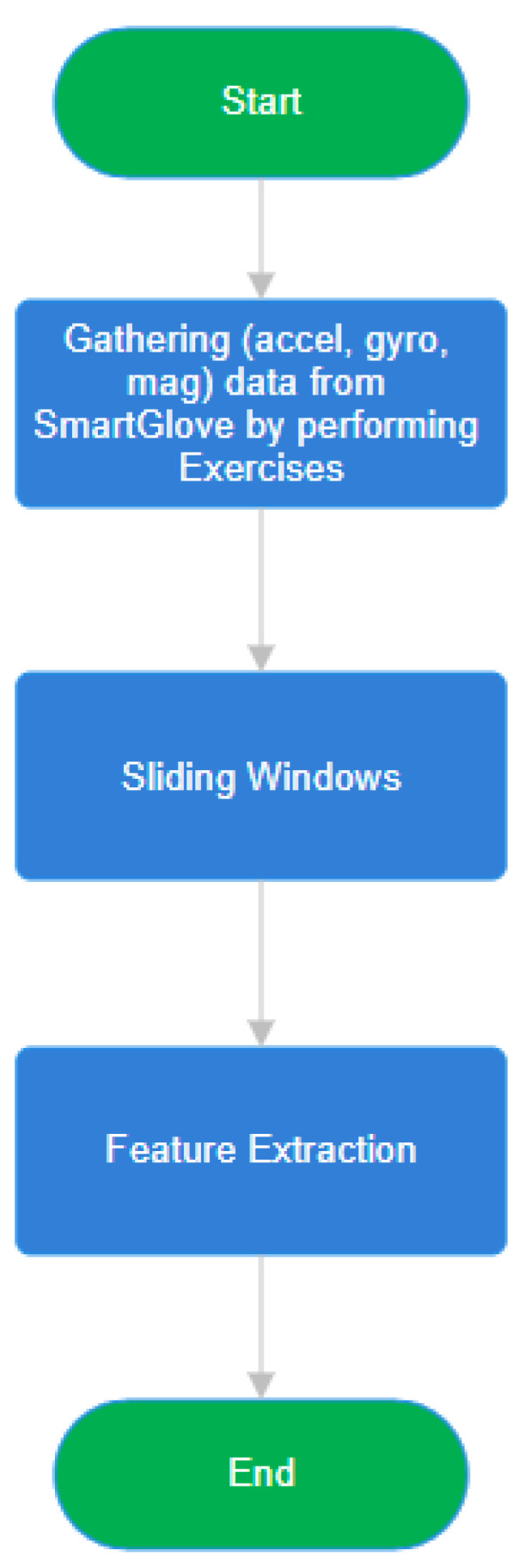
Multi-domain feature extraction process. Flowchart summarizing the complete pipeline that transforms raw sensor signals from the accelerometer, gyroscope, and magnetometer into statistical, frequency-domain, and nonlinear dynamic features used for further analysis.

For the tri-axial accelerometer, gyroscope, and magnetometer data, the magnitude of the signal vector was first calculated for each time point as

(1) $$d=\sqrt{x^{2}+y^{2}+z^{2}}$$

and the subsequent features were then extracted from this combined magnitude signal to produce a single value per feature. The flowchart of Figure 5 illustrating the multi-domain feature extraction process from raw sensor data, including statistical, energy, frequency-domain, and dynamic/nonlinear features.

### 2.4. Feature Selection Methodology

The feature selection process was applied to a dataset derived from a cohort of 14 patients with Parkinson’s disease. Data were collected for each patient in both the practical OFF and ON medication states. The ON state assessment was conducted 1–1.5 h after medication intake. To generate a sufficient number of instances for robust model development from the kinematic time-series, the feature extraction process (Section 2.3) with 1-s windows and 50% overlap was used. This yielded a final dataset of 251, 357, 427, and 333 feature vectors for Exercises 0, 1, 2, and 3, respectively. The following methodology was used to identify the most discriminative features within this dataset.

Feature selection is a critical step in developing accurate and interpretable machine learning (ML) models for PD detection. In this study, a multi-method scoring approach was applied to identify the most informative features that differentiate between pre-medication and post-medication states. Instead of relying on a single method, three complementary techniques were employed, and their outputs were combined into a composite score.

#### 2.4.1. Multi-Method Scoring Approach

To accurately evaluate the value of each characteristic for differentiating between pre- and post-medication states, a multi-angled scoring approach was adopted. For each feature, scores for three different aspects, such as statistical significance, importance based on the model, and variance contribution, were computed.

- Statistical Significance (Paired T-Test): The paired T-Test was then carried out on measurements preceding and succeeding medication on each dimension. To make the number significant, the $p_{\mathrm{value}}$ was transformed into a score using the following formula:
  (2) $${\mathrm{score}}_{T_{\mathrm{test}}}=-\log_{10}\left(p_{\mathrm{value}}+\epsilon \right)$$
  Here, $e=1\times 10^{-10}$ is a very small constant introduced for avoiding numerical instability arising from a $p_{\mathrm{value}}$ of zero. This mapping makes variables with lower $p_{\mathrm{value}}$ have exponentially large scores, hence indicating more considerable statistical evidence against the null hypothesis ([33]).
- **Model-Based Importance (Random Forest)**: The Random Forest classifier was built such that it could distinguish between the two different states. The importance of a specific feature was computed using the average reduction in Gini impurity across all trees in the ensemble. Gini impurity is a measure of the purity of a node, and the importance is given by the weighted cumulative reduction in node impurity, divided by the probability of the node, and averaged across trees [34]. The calculation was performed as follows: For a single tree *T*, the importance of feature *f* is the sum of its Gini impurity decreases across all nodes where it is used for splitting:
  (3) $${\mathrm{Importance}}_{T}\left(f\right)=\sum_{n\in S\left(T,f\right)}\Delta \mathrm{Gini}\left(n,f\right)$$
  where $S\left(T,f\right)$ is the set of all nodes in tree *T* split by feature *f*, and $\Delta \mathrm{Gini}\left(n,f\right)$ is the decrease in Gini impurity at node *n* achieved by splitting on *f*. The importance is then averaged across all $N_{\mathrm{trees}}$ trees in the forest to obtain the final score:
  (4) $${\mathrm{score}}_{RF}\left(f\right)=\frac{1}{N_{\mathrm{trees}}}\sum_{T=1}^{N_{\mathrm{trees}}}{\mathrm{Importance}}_{T}\left(f\right)$$
  Features with a higher score ${\mathrm{score}}_{RF}\left(f\right)$ are more influential in the model’s decision-making process.
- **Variance Contribution Score (PCA)**: Principal Component Analysis (PCA) was performed on the standardized data set. The contribution of a individual original feature towards the principal components (PCs) was measured by adding its absolute loadings across all PCs. The loading of a feature onto a PC is its correlation with the specific component [35]. The score ${\mathrm{score}}_{\mathrm{PCA}}$ was computed as follows:
  (5) $${\mathrm{score}}_{\mathrm{PCA}}=\sum_{i}\left|{\mathrm{loading}}_{i}\right|$$
  Here, the ${\mathrm{loading}}_{i}$ is the feature weight corresponding to the *i*-th principal component. This cumulative sum provides the total impact of the feature on the total variance tackled by the Principal Component Analysis (PCA) model [36].

#### 2.4.2. Composite Score Calculation

To reduce these unrelated measurements into one unified measure of feature prominence, the scores were amalgamated. Each score was first normalized between [0,1] using min-max normalization for comparability across scores. A clear composite score was then calculated as a weighted average of the normalized scores:

(6) $$\mathrm{Composite}\;\mathrm{Score}={0.4\times \mathrm{score}}_{T_{\mathrm{test}}}+0.3\times {\mathrm{score}}_{RF}+0.3\times {\mathrm{score}}_{\mathrm{PCA}}$$

This weighting approach was chosen so that features which are highly statistically significant (by the T-test) are given priority, first, while data from model-based learning (Random Forest) and variance-based analysis (PCA) are incorporated cautiously

#### 2.4.3. Selection of Best Features

Features were ranked according to their composite score, and the top-performing ones were selected for subsequent model development. Representative high-ranking features includes the following:

- RMSSD from the gyroscope during Exercise 2 (score: 0.663);
- Lyapunov Exponent (score: 0.644);
- Higuchi Fractal Dimension (score: 0.612).

These selected features served as the foundation for later predictive modeling and analysis, aiming to capture the most relevant motor signal characteristics associated with PD symptoms [37,38]. In Table 2 the top 20 features are presented.

Following the ranking by composite score, the top 80% of features were retained for the subsequent model development and feature construction process.

### 2.5. Grammatical Evolution Preliminaries

The Grammatical Evolution procedure can be considered a genetic algorithm, where the chromosomes are sets of randomly chosen integers. These integers represent production rules of the provided BNF grammar [39]. BNF grammars are usually expressed as sets having the form $G=\left(N,T,S,P\right)$, where

- The set *N* contains the non-terminal symbols of the grammar.
- The set *T* includes the terminal symbols of the grammar.
- *S* denotes the start symbol of the grammar, with S∈N.
- *P* is the set of production rules of the grammar.

**Figure 6:**
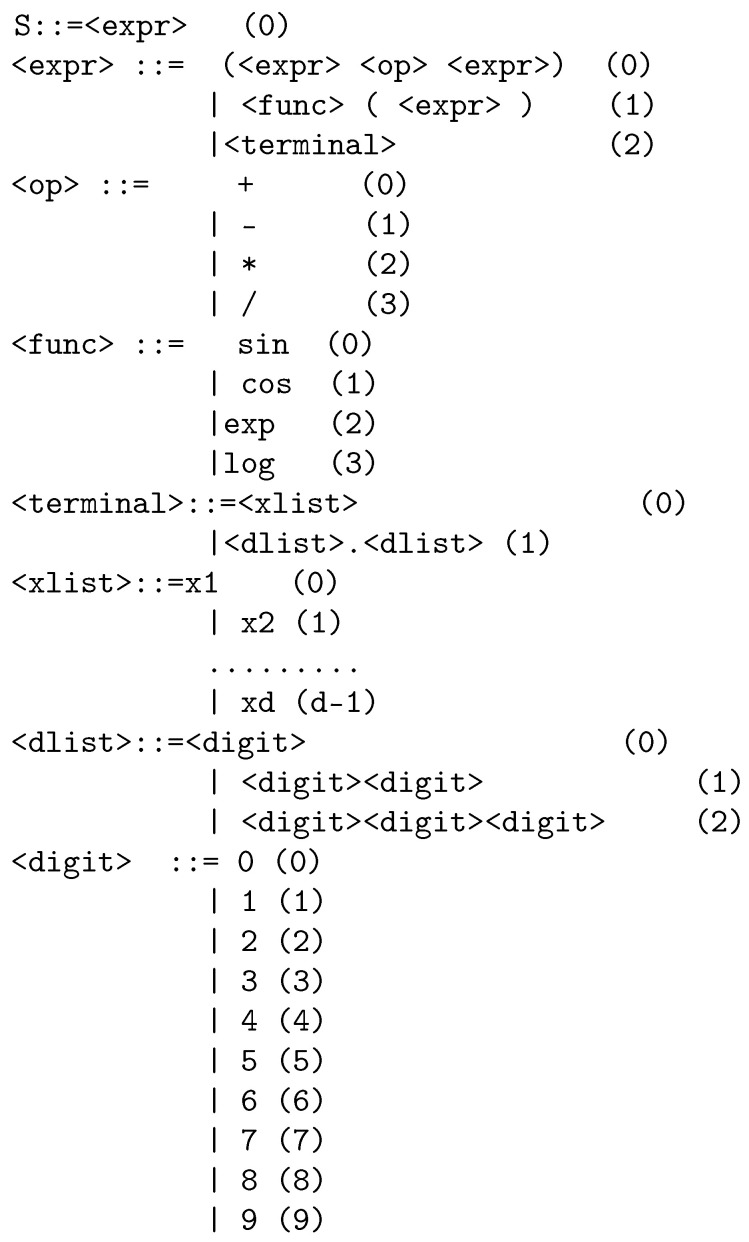
Example of an extended Backus–Naur Form (BNF) grammar used in Grammatical Evolution. The diagram outlines the structure of production rules applied to generate new mathematical expressions from existing features, enabling the automatic construction of artificial variables through evolutionary search.

The Grammatical Evolution procedure utilizes an extended version of the initial BNF grammar by adding enumeration in the production rules. As an example of an extended BNF grammar, consider the grammar shown in Figure 6. The notation < > is used to enclose the nonterminal symbols of the grammar. The value *d* denotes the dimension of the input data. The Grammatical Evolution procedure starts from the start symbol of the program and gradually it creates valid programs in the underlying language, using the production rules of the grammar. The general scheme of the production procedure has as follows:

- **Get** the next element V from the chromosome that is under processing.
- **Select** the next rule using the equation Rule = V mod NR. The symbol NR denotes the number of production rules for the under processing nonterminal symbol.

A full working example of the production procedure is the chromosome

$$x=\left[9,8,6,4,16,10,17,23,8,14\right]$$

with d=3. After a series of steps, the function $f\left(x\right)=x_{2}+\cos\left(x_{3}\right)$ is produced for the grammar of Figure 6. The production steps are shown in Table 3.

### 2.6. The Feature Construction Method

**Figure 7:**
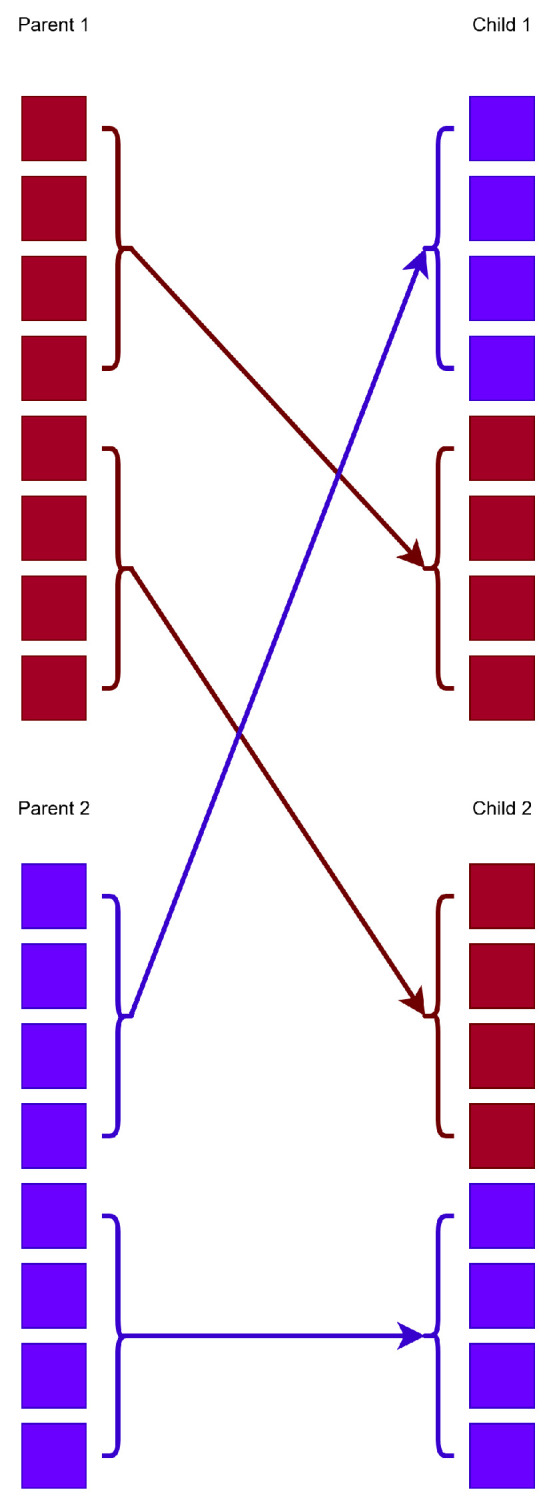
One-point crossover operation in the Grammatical Evolution procedure. Schematic representation showing how two parent chromosomes exchange genetic material to produce offspring solutions during the evolutionary optimization step. The red colors indicate the elements of the first chromosome that participate in the crossover procedure and the blue colors represent the second chromosome.

The technique of feature construction produces artificial features for classification or regression problems as nonlinear transformations of the original ones. The new features are evaluated using a machine learning technique, such as artificial neural networks [40,41] or a Radial Basis Function (RBF) networks [42,43]. This method was presented initially in [44]. Also, this method was used in many areas, such as Spam Identification [45], Fetal heart classification [46], signal processing [47,48] etc. The process has the following steps:

1. **Initialization** step.
  (a) **Obtain** the train data for the current problem. The train data have *M* pairs $\left(x_{i},t_{i}\right),\;i=1\ldots M$. The dimension of each vector $x_{i}$ is *d*. The values $t_{i}$ are the expected outputs for each pattern.
  (b) **Define** the parameters of the genetic algorithm: $N_{g}$ stands for the number of allowed generations, $N_{c}$ represents the number chromosomes, $p_{s}$ defines the selection rate and $p_{m}$ the mutation rate.
  (c) **Set** as $N_{f}$ the number of features that will construct the Grammatical Evolution procedure.
  (d) **Initialize** the chromosomes in the population. Each chromosome is considered as a set of randomly chosen positive integers.
  (e) **Set**k=1, as the generation counter.
2. **Genetic step**
  (a) **For $i=1,\ldots ,N_{c}$ do**
    - **Produce** using the Grammatical Evolution procedure a set of $N_{f}$ artificial features for the corresponding chromosome $g_{i}$. These features are produced using the grammar of Figure 6.
    - **Transform** the original dataset using the previously constructed features. The new training set is denoted as $\left(x_{g_{i},j},t_{j}\right),\;j=1,\ldots M$
    - **Train** a machine learning model denoted as *C* using the new training set. The fitness $f_{i}$ for the corresponding chromosome is defined as follows:
      (7) $$f_{i}=\sum_{j=1}^{M}{\left(C\left(x_{g_{i},j}\right)-t_{j}\right)}^{2}$$
      In the proposed method, the RBF network is used as the machine learning model, since the time required for its training is significantly lower than other models, such as neural networks.
    - **Perform** the selection procedure. During this procedure, the chromosomes are firstly sorted according to their fitness values and the best $\left(1-p_{s}\right)\times N_{C}$ of them are copied intact to the next generation. The rest of the chromosomes will be substituted by new chromosomes produced during the procedures of crossover and mutation.
    - **Perform** the crossover procedure. During this procedure $p_{s}\times N_{c}$, new chromosomes are produced. For every pair of $\left(\tilde{z},\tilde{w}\right)$ of new chromosomes, two distinct chromosomes $\left(z,w\right)$ are selected from the current population, which is performed using tournament selection. The new chromosomes are produced from the old ones using the one-point crossover procedure, which is graphically outlined in Figure 7.
    - **Perform** the mutation procedure: for each element of each chromosome, a random number $r\in \left[0,1\right]$ is selected. The underlying element is altered randomly when $r\leq p_{m}$.
  (b) **EndFor**
3. **Set** k=k+1
4. **If** $k\leq N_{G}$ goto **Genetic** Step, else terminate the process and obtain the best chromosome $g^{*}$ with the lowest fitness value.
5. **Transform** the corresponding test set, using the $N_{f}$ features obtained for chromosome $g^{*}$.
6. **Apply** a machine learning model to the constructed test set and report the associated test error.

**Figure 8:**
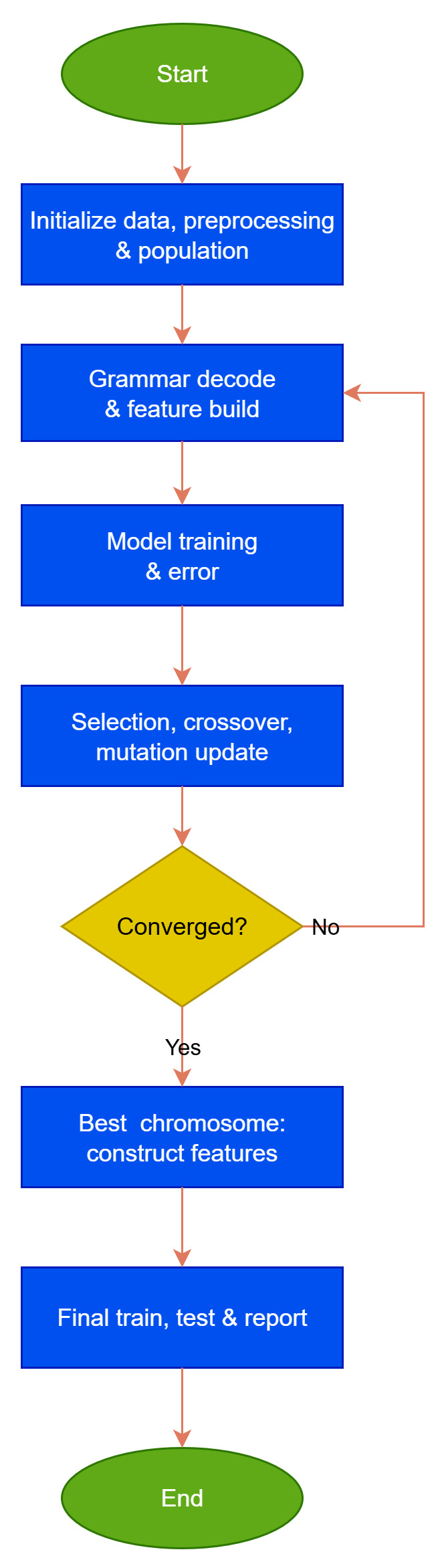
Flowchart of the genetic algorithm applied for feature construction. Step-by-step depiction of the Grammatical Evolution workflow, from population initialization and evaluation to selection, crossover, and mutation, leading to optimized artificial features.

A flowchart of the previous algorithm is shown in Figure 8.

**Figure 9:**
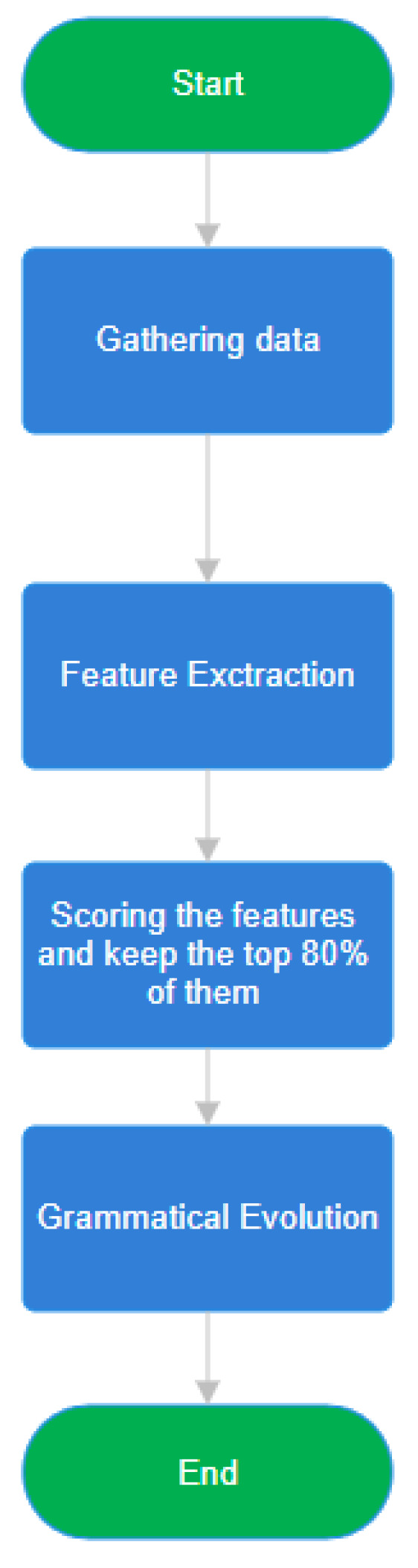
End-to-end methodological framework for Parkinson’s Disease motor symptom analysis. Comprehensive overview of the entire SmartGlove pipeline—from sensor-based data acquisition and preprocessing to feature selection, Grammatical Evolution, and final machine-learning classification.

The flowchart that describes the proposed methodology for PD motor symptom analysis, from data acquisition using the SmartGlove to feature construction and classification is shown in Figure 9.

## 3. Results

The machine learning models were applied to a binary classification task to distinguish between the pre-medication (OFF) and post-medication (ON) states. The code used in the experiments was code in the C++ programming language, and for the optimization methods, the freely available Optimus programming tool was incorporated [49]. The experiments were conducted on machine with 128 GB of ram, running the Debian Linux operating system. Each experiment was executed 30 times and the average classification error was measured and depicted in the related tables and graphs. Also, the 10-fold cross validation technique was incorporated for the validation of the experimental results. Table 4 presents the distribution of patterns into categories for each data set. The values for the parameters of the proposed method are depicted in Table 5.

In the experimental tables, the following notation is used:

1. The column DATASET represents the performed exercise.
2. The column RBF stands for the application of an RBF neural network [42,43] with 10 processing nodes on the corresponding dataset.
3. The column GEN represents the application of a genetic algorithm [50] on the training process of a neural network with 10 processing nodes.
4. The column PCA stands for the application of the PCA method [51,52,53] to construct two artificial features from the original ones. Afterwards, a neural network with 10 processing nodes trained using the BFGS method is applied on the new datasets.
5. The column NNC stands for the application of a neural network constructed with Grammatical Evolution [54] on the corresponding dataset.
6. The column GENCLASS represents the usage of a method that constructs classification rules using Grammatical Evolution [55].
7. The column FC2 is used to represent the application of a genetic algorithm to train a neural network on the dataset produced by the construction of two artificial features using the feature construction method.
8. The column FC3 is used to represent the application of a genetic algorithm to train a neural network on the dataset produced by the construction of three artificial features using the feature construction method.
9. The column FC4 is used to represent the application of a genetic algorithm to train a neural network on the dataset produced by the construction of four artificial features using the feature construction method.
10. The row AVERAGE represents the average classification error for all datasets and the corresponding method.

**Figure 10:**
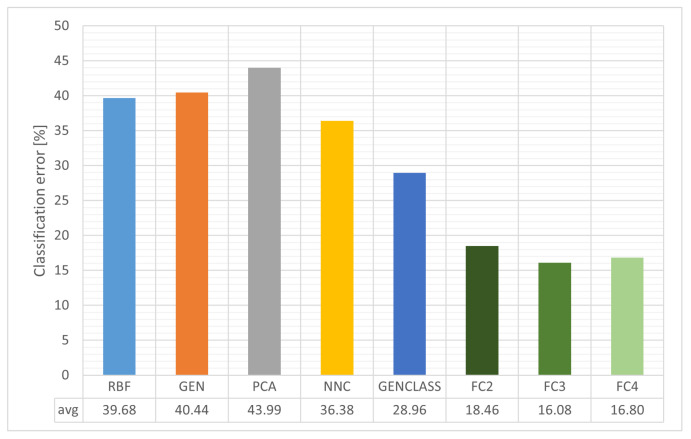
Average classification error across all evaluated methods. Bar plot comparing the performance of different machine-learning approaches, showing that the proposed feature-construction models (FC2–FC4) consistently achieved the lowest classification errors, confirming the effectiveness of the Grammatical Evolution approach.

Table 6 reports classification error per exercise and per feature/model family, where lower values are better. The proposed Feature Construction approach (columns FC2, FC3, and FC4) consistently yields lower errors than classical baselines such as PCA, RBF, NNC, GEN, and GENCLASS, indicating that the constructed descriptors capture the discriminative motion patterns more effectively. Based on the overall average, FC3 is the strongest among the proposed variants, achieving the lowest mean error, with FC4 very close behind and FC2 also competitive. In EXERCISE 0, the Feature Construction variants clearly dominate, with FC3 providing the best balance between expressiveness and generalization, FC4 performing nearly as well, and FC2 remaining stable. In EXERCISE 1, the picture is similar: FC2–FC4 retain a lead over classical pipelines and FC3 acts as the “sweet spot,” while FC4 is the slightly more aggressive variant and FC2 the conservative yet reliable option. In EXERCISE 2, where separability is more challenging, the FC columns keep the error noticeably lower than the classical models and FC3 generally maintains an edge, although FC4 occasionally approaches or overlaps within small margins. In EXERCISE 3, the same trend holds, with FC2, FC3, and FC4 achieving lower errors and FC3 preserving the overall lead among the proposed methods. Overall, Table 5 underscores that representation quality precedes classifier complexity, when features are properly constructed, even relatively simple decision rules can achieve consistently low error. In practice, orchestrating Feature Construction with linear or RBF-based decision layers or adopting a hybrid design where feature construction is followed by mild dimensionality reduction preserves the information advantage while further controlling noise. The average results for this table are also outlined graphically in Figure 10.

Also the precision values for all exercises and machine learning methods are outlined in Table 7. Similarly, the recall values are depicted in Table 8.

**Figure 11:**
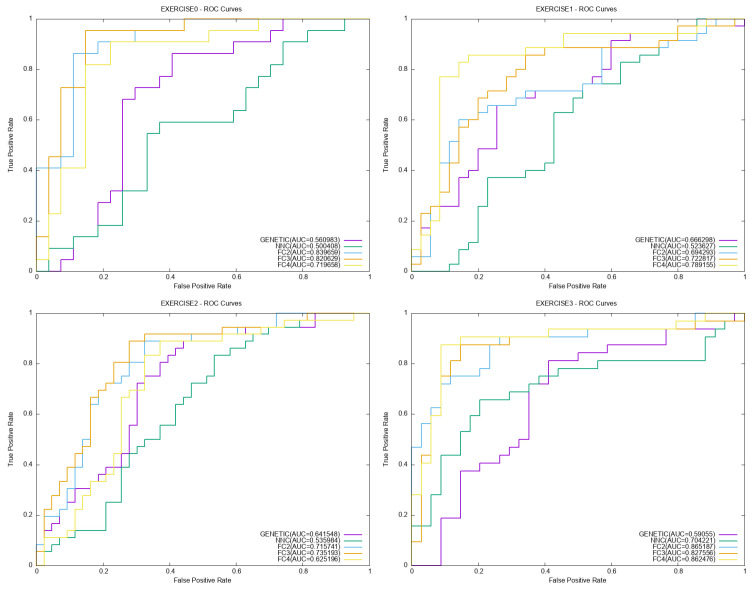
Indicative plots for the ROC curves, involving the genetic algorithm, the NNC method and the proposed feature construction technique.

**Figure 12:**
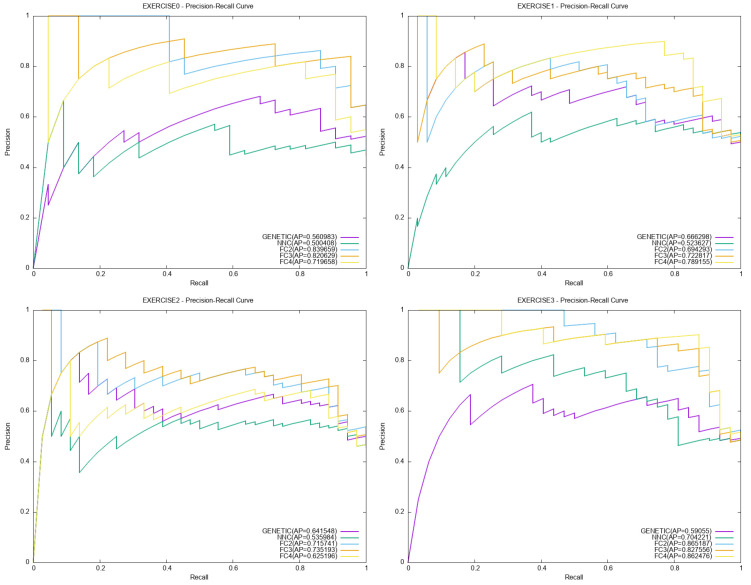
Indicative plots for the PR curves.

Moreover, indicative plots for ROC and PR performance curves are outlined in Figure 11 and Figure 12 respectively.

## 4. Discussion

### 4.1. Comparison of Motor Exercises Study

The performance of our models was inconsistent across the four exercises, but it also gave us important information about which motor activities produce the most discriminative data for distinguishing pre- and post-medication conditions. The easiest task for classification was Exercise 0 (Resting Tremor Observation) for all FC models, which produced the best results with the least error rates of around 10–11%. The excellent performance here implies that resting-state kinematics, almost certainly including subtle tremor and rigidity, are greatly and reliably impacted by medication. The relative ease of such a task ensures that, to some extent, “voluntary noise” due to move decisions is minimized, such that the model isolates essential pathological signatures that were reliably modulated by medicine. On the other hand, Exercises 2 and 3 (Finger Tapping and Hand Opening–Closing), as tests for bradykinesia and rigidity, also had good results but with slightly elevated error rates. The rapid, repeated character of these tests for generating rich dynamic and nonlinear data, and the saliency of features such as the Root Mean Square of Successive Differences (RMSSD), and the Lyapunov Exponent of the gyroscope, highlight that the smoothness and predictability of motion is a chief biomarker. Finally, Exercise 1 (Postural Tremor and Coordination) was the most difficult for our models. The complexity of the task, with more complex planning of motor actions, can create data with higher inter-subject variability or can involve neurobiological circuitry that is less reliably impacted by medication in the short term. In conclusion, while all of these exercises contributed, the most reliable information for the production of discriminative features in such a specific classification task were the resting tremor observation and specifically conducted bradykinesia tests.

### 4.2. Effectiveness of the Multi-Method Feature Scoring System

One of the key contributions of the present work is the composite feature scoring system, whereby it was successful in finding a sparse set of highly relevant biomarkers without prior recourse to a particular ML algorithm. The power of such an approach is in the tripartite validation, whereby it combined statistical significance, model-driven importance, and variance contribution. The first, the statistical T-test, ensured that all chosen features had a significant mean shift between pharmacological states at the population scale, a prerequisite for a useful biomarker. The second, the Random Forest importance, assessed each feature’s informativeness in a nonlinear, ensemble learning framework, highlighting those that were most effective in splitting the data. The third, PCA variance contribution, ensured that chosen features captured prominent patterns in the dataset, optimizing for non-redundancy. Empirical verification of such a strategy is in the composition of the top-ranked features, whereby they were dominated by nonlinear dynamic measures such as the Lyapunov exponent and Higuchi fractal dimension. This suggests that the system successfully ranked features that described the complexity and chaos of PD motor control as most important, something these sorts of simplistic statistical mean-based methods oftentimes fail to capture. The reason why these very features subsequently became the foundation of the highly successful Grammatical Evolution process is that it empirically identifies that the scoring system was extraordinarily successful in reducing the raw feature space to the most physiologically relevant candidates of biomarkers, with the direct consequence being the dramatic reduction in classification error. Hence, this is not so much a victory of the ultimate model, but rather a direct consequence of such a rigorous, multi-faced scoring system that revealed these data-driven biomarkers most relevant to the patient pharmacological state.

### 4.3. Interpretability and Biomarker Explainability

An additional perspective of the present work lies in the interpretability of the extracted and constructed features. Although the Grammatical Evolution framework operates in a data-driven way, the resulting artificial features were combinations of statistically significant and physiologically meaningful descriptors. Many of the top-ranked inputs in the feature scoring phase—such as the Lyapunov exponent, the Higuchi fractal dimension, and the Root Mean Square of Successive Differences (RMSSD)—are directly linked to the smoothness, stability, and complexity of movement, all of which are well-established indicators of motor impairment in Parkinson’s disease.

In this sense, these variables can be viewed as digital biomarkers that reflect motor control degradation and the effect of medication on movement regularity. To further support interpretability, the contribution of each feature to model output was examined through its normalized importance during classification, showing that nonlinear dynamic descriptors consistently dominated the decision space. This correspondence between physiologically interpretable features and predictive importance indicates that the model’s decision process aligns with known pathophysiological mechanisms of Parkinsonian movement. Future versions of this work will include explicit explainability analyses (e.g., SHAP or LIME) to visualize feature contributions at the single-subject level, thereby reinforcing the transparency of the predictive framework and supporting clinical translation.

### 4.4. Limitations and Future Work

Despite the successful findings, the current study is limited by specific drawbacks that define clear paths for future research. A primary limitation of this study is the partial utilization of the SmartGlove’s full sensor suite. Our analysis was deliberately focused on the data from the inertial measurement unit (IMU). This focus allowed us to develop and validate our core methodological contribution, the multi-faceted feature scoring and Grammatical Evolution framework, on a rich but well-established kinematic data modality for assessing gross motor symptoms like tremor and bradykinesia. While the IMU data was highly informative, we acknowledge that the flex sensors and finger-to-palm contact sensors were not utilized. These sensors have the potential to provide direct, quantitative measures of rigidity (e.g., resistance to passive movement) and finer aspects of bradykinesia (e.g., range and velocity of individual finger flexion, completeness of finger-tapping contacts) that are currently only inferred from IMU kinematics. To overcome this limitation, our immediate future work includes the full integration of data from all available sensors into the feature extraction and modeling pipeline, which will enable a more holistic and precise quantification of hand motor function in Parkinson’s disease.

Additionally, the range of this research was limited by a modestly sized pool of participants. To legitimize the reliability and portability of the thus derived features, a larger and more heterogeneous dataset is required that would encompass patients in various stages of PD, in association with various age groups, illness duration, and treatment regimens. It would make the feature selection process statistically more robust and legitimize that the thus determined biomarkers would represent the whole PD population, rather than only being unique to some subgroup. To overcome such identified limitations and extend our findings further, our subsequent research will follow multiple avenues. The near term is the full integration of data from the flex and contact sensors into the feature extraction protocol, thus generating a unified multi-modal dataset more comprehensively capturing the phenomenology of hand motion. Concurrently, we initiate a large-scale clinical validation study for the purpose of collecting data from a much larger population of subjects in diverse clinical settings. This project shall allow us not only to verify the efficacy of our present features but also examine the feasibility of developing models for patient stratification by disease severity or for the prediction of individual responses to therapy. Our long-term aim is to develop this methodology into a useful, sustained, at-home, practical monitor, providing continuous, objective information for personalized therapeutic strategies for PD.

## 5. Conclusions

This study successfully formulated a rigorous methodology for the unbiased assessment of motor symptoms in PD, finding that advanced feature engineering is necessary for accurate classification. The main finding is that the generation of artificial features by Grammatical Evolution drastically outperforms conventional machine learning techniques operating with raw data. By generating a short list of two to four strongly discriminative features, we achieved a significant reduction in classification error, with the best performing model reaching the feature construction model.

The findings of our research contributed new meaningful information to the dataset. The study indicated that few of the motor functions, specifically observation of the resting tremor and execution of directional bradykinesia testing, create the most discriminable kinematics for ascertaining drug effectiveness. Additionally, the multi-modal feature scoring system revealed meaningful potential for discerning the most important biomarkers, of which the nonlinear dynamics as well as signal complexity became prominent features of motor impairment for PD.

Future studies will extend beyond the current boundaries by utilizing data from the unused contact and flex sensors, and by validating the system in a larger and more heterogeneous patient population. The long-term vision is to build such methodology into a practical, user-friendly in-home, real-time monitor that gives clinicians empirical data to personally guide therapeutic interventions and achieve better long-term patient outcomes.

## Figures and Tables

**Table 1:** Table of characteristics for movement analysis in Parkinson’s disease.

Category	Characteristics	Purpose	Clinical Significance
Central Tendency	Mean, Median	Median Measures the central value of the signal	Detection of bradykinesia (slowed movements)
Dispersion	Standard deviation, Variance, IQR, QD	Quantifies the spread of data	Identifies motion variability (e.g., gravity, tremor)
Range	Minimum/Maximum value, Range	Records extreme values	Assessment of range of motion reduction in patients with PD
Distribution Shape	Skewness, Kurtosis	Describes asymmetry/peakedness of distribution	Correlation with irregular motor patterns
Variability	Coefficient of Variation (CV), MAD, RMSSD	Normalized measures of dispersion	Variability differentiation before after the treatment
Energy	Total energy, Absolute Energy, RMS	Measures signal intensity	Correlation with hypokinesia (reduced motor energy)
Logarithmic Energy	Log Energy	Enhances subtle energy changes	Detection of small changes
Spectral	Spectral Entropy, Centroid	Analyzes frequency distribution	Localization of tremor and rhythmic abnormality
Roll-off	85% Roll-off	Frequency band where	
85% of the power is concentrated	Characterization of tremor bandwidth		
Dominant Frequency	Dominant Frequency	Identification of peak frequency	Detection of Parkinson’s tremor (4–6 Hz)
Spectral Shape	Flatness, Flux, Variability, Dispersion	Quantifies the stability of the spectrum	Unstable tremors vs. rhythmic movements
Dynamics	Lyapunov Exponent, Sampling Entropy	Evaluates the chaos/regularity of the system	Degeneration of motor control in Parkinson’s
Fractal	Dimension Higuchi	Measures the complexity of the signal	Loss of fine motor control

**Table 2:** Top 20 features scored.

Exercise	Sensor	Feature Name	Composite Score
2	gyro	rmssd	0.663
2	gyro	lyapunov_exponent	0.644
2	gyro	higuchi_fractal_dimension	0.612
2	gyro	range	0.573
3	acce	higuchi_fractal_dimension	0.469
3	gyro	max	0.452
3	acce	lyapunov_exponent	0.437
2	gyro	spectral_rolloff	0.433
2	acce	sample_entropy	0.422
0	magn	lyapunov_exponent	0.406
0	magn	spectral_variability	0.399
0	magn	higuchi_fractal_dimension	0.399
0	magn	range	0.381
3	magn	rmssd	0.376
0	gyro	rmssd	0.375
2	gyro	std	0.373
3	acce	spectral_flux	0.367
1	gyro	lyapunov_exponent	0.365
1	gyro	rmssd	0.361
1	gyro	max	0.358

**Table 3:** A series of production steps for the example chromosome.

String	Chromosome	Operation
<expr>	9, 8, 6, 4, 16, 10, 17, 23, 8, 14	9mod3=0
(<expr><op><expr>)	8, 6, 4, 16, 10, 17, 23, 8, 14	8mod3=2
(<terminal><op><expr>)	6, 4, 16, 10, 17, 23, 8, 14	6mod2=0
(<xlist><op><expr>)	4, 16, 10, 17, 23, 8, 14	4mod3=1
(x2<op><expr>)	16, 10, 17, 23, 8, 14	16mod4=0
(x2+<expr>)	10, 17, 23, 8, 14	10mod3=1
(x2+<func>(<expr>))	17, 23, 8, 14	17mod4=1
(x2+cos(<expr>))	23, 8, 14	23mod2=2
(x2+cos(<terminal>))	8, 14	8mod2=0
(x2+cos(<xlist>))	14	14mod3=2
(x2+cos(x3))		

**Table 4:** Distribution of patterns for each dataset.

DATASET	CLASS-0	CLASS-1
EXERCISE0	251	241
EXERCISE1	357	343
EXERCISE2	427	364
EXERCISE3	333	328

**Table 5:** The values for the parameters for the current work.

PARAMETER	MEANING	VALUE
$N_{g}$	Number of maximum allowed generations.	500
$N_{c}$	Number of chromosomes	500
$p_{s}$	Selection rate	0.10
$p_{m}$	Mutation rate	0.05
*H*	Number of processing nodes for neural network	10

**Table 6:** Experimental results for various exercises.

DATASET	RBF	GEN	PCA	NNC	GENCLASS	FC2	FC3	FC4
EXERCISE 0	40.86%	39.63%	44.70%	32.57%	25.52%	11.39%	11.06%	10.35%
EXERCISE 1	38.65%	47.61%	47.35%	42.07%	29.98%	20.16%	16.08%	14.84%
EXERCISE 2	37.57%	35.33%	40.46%	35.01%	31.34%	22.50%	19.78%	22.10%
EXERCISE 3	41.64%	39.17%	43.46%	35.88%	29.00%	19.79%	17.39%	19.91%

**Table 7:** Precision values for all methods participated in the experiments.

DATASET	RBF	GEN	PCA	NNC	GENCLASS	FC2	FC3	FC4
EXERCISE 0	0.589	0.603	0.559	0.673	0.761	0.885	0.889	0.898
EXERCISE 1	0.62	0.496	0.581	0.595	0.713	0.795	0.837	0.851
EXERCISE 2	0.616	0.726	0.582	0.646	0.69	0.778	0.803	0.78
EXERCISE 3	0.583	0.608	0.566	0.642	0.715	0.803	0.829	0.803

**Table 8:** Recall values for all methods used in the conducted experiments.

DATASET	RBF	GEN	PCA	NNC	GENCLASS	FC2	FC3	FC4
EXERCISE 0	0.588	0.603	0.559	0.689	0.75	0.883	0.885	0.893
EXERCISE 1	0.619	0.766	0.605	0.626	0.711	0.797	0.839	0.854
EXERCISE 2	0.626	0.646	0.589	0.653	0.674	0.771	0.801	0.78
EXERCISE 3	0.583	0.608	0.567	0.652	0.713	0.801	0.827	0.802

## Data Availability

The raw data supporting the conclusions of this article will be made available by the authors on request.
